# Application of real-time simultaneous amplification and testing method to accurately and rapidly detect extra-pulmonary tuberculosis

**DOI:** 10.1186/s12879-020-05036-0

**Published:** 2020-04-22

**Authors:** Tongxin Li, Tao Shi, Ying Sun, Kan Zhou, Zhenggu Huang, Pengsen Wang, Ming Luo, Xiaoping Nie, Guoqiang Yang, Yu Chen, Yaokai Chen

**Affiliations:** 1 Central Laboratory, Chongqing Public Health Medical Center, No. 109, Baoyu Road, Shapingba District, Chongqing, 400036 China; 2 Department of Orthopedics, Tianjin First Center Hospital, No.24, Fukang Road, Nankai District, Tianjin, 300192 China; 3 Department of Respiratory, Tianjin Hexi Hospital, No. 40, Qiongzhou Road, Hexi District, Tianjin, 300202 China; 4 Department of Clinical Laboratory, Chongqing Public Health Medical Center, No. 109, Baoyu Road, Shapingba District, Chongqing, 400036 China; 5 Department of Orthopedics, Chongqing Public Health Medical Center, No. 109, Baoyu Road, Shapingba District, Chongqing, 400036 China; 6 Medical Records Room, Chongqing Public Health Medical Center, No. 109, Baoyu Road, Shapingba District, Chongqing, 400036 China

**Keywords:** Extra-pulmonary, *Mycobacterium tuberculosis*, Simultaneous amplification and testing, Specificity, Sensitivity

## Abstract

**Background:**

This study aimed to establish and evaluate a simultaneous amplification and testing method for detection of extra-pulmonary tuberculosis (EPTB).

**Methods:**

From January 2016 and December 2017 the pus or surgical excision from the lesions of inpatients admitted from Chongqing Public Health Treatment Center were collected. According to the clinical diagnosis, the samples were divided into two groups including EPTB (Group A) and other diseases excluded from tuberculosis diseases (Group B). Simultaneous detection of *Mycobacterium tuberculosis* (MTB) used Roche culture method, liquid culture method and simultaneous amplification and testing (SAT) method. The sensitivity and specificity of the SAT method were compared with culture methods and clinical diagnosis of EPTB.

**Results:**

For 433 EPTB specimens and 49 non-TB specimens, the simultaneous amplification and testing tuberculosis (SAT-TB) results correlated with 80.5% (388/482 specimens) of the culture assay results. The sensitivity, specificity, and positive and negative predictive values of the SAT-TB test for the diagnosis of EPTB were 83.6, 79.4, 59.4, and 93.0%, respectively, compared to culture methods. Compared with the clinical diagnosis of patients, the sensitivity and specificity of the SAT-TB test were 41.6 and 100%, respectively, the cultures test were 29.3 and 98.0%.

**Conclusions:**

SAT test is a simple and rapid test with high specificity which may enhance the detection of EPTB. SAT-TB is a higher clinical diagnosis value for EPTB in clinical microbiology laboratories.

## Background

Tuberculosis (TB) is a chronic infectious disease that endangers human health for a long time. Its clinical manifestations are extremely diverse, with 80% of TB infections occurring in the lungs and other sites like cervical lymphadenitis, meninges, peritoneum, intestines, skin, and bones [1]. According to the WHO Global Tuberculosis Report 2015 32,000 cases of extra-pulmonary tuberculosis (EPTB) were diagnosed in nearly 530,000 new cases in China in 2014 [2]. In 2018 there were an estimated 10.0 million people fell with TB and 1.2 million death [3]. It is difficult to diagnose EPTB due to its extensive lesions and complicated clinical manifestations.

Rapid and accurate laboratory testing technology plays an important role in assisting the early diagnosis of EPTB in clinical practice. Simultaneous Amplification and Testing (SAT) is one of the molecular biological detection methods used by the tuberculosis laboratory in China in recent years. It is mainly used to detect MTB in sputum to assist diagnosis [4–7]. However, SAT has rarely been used to test EPTB specimens and therefore, its sensitivity and specificity for EPTB diagnosis are still not clear. This study aimed to evaluate the clinical accuracy of SAT in the diagnosis of EPTB by comparing sensitivity, specificity, positive predictive value and negative predictive value of both SAT and culture methods in detecting EPTB specimens.

## Methods

### Patients

Four hundred eighty-two cases of inpatients with EPTB, who were hospitalized in Chongqing Public Health and Medical Center from January 2016 to December 2017, were included in this study. Among them, there were 433 cases of confirmed EPTB patients (Group A) and 49 cases of non-TB patients (Group B). Of all the 482 patients, 259 were male and 223 were female. The average age of all 482 patients was 35.5 years, ranging from 7 years to 79 years.

### Specimen collection and pretreatment

Specimens (pus and excised tissues) were collected during surgical procedures and stored at low temperature (4–8 °C). Then, the specimens were decontaminated using the N-acetyl-L-cysteine (NALC)–NaOH method [8]. The processed sediment was washed using a sterile 0.9% NaCl solution, re-suspended in 1.5 ml sterile 0.9% NaCl solution and then equally divided into three thirds.

### Specimen separation and culture

One of the three thirds was then centrifuged and the sediment was inoculated in both Bactec MGIT 960 system (Bacton Dickinson and Company, MD) and neutral Roche medium. The culture was regarded as positive if one or both of the above two culture methods produced positive results.

### Specimen tuberculosis simultaneous amplification and testing (TB-SAT) detection

Another third was centrifuged at 13000 rpm for 5 min, and the supernatant were discarded. The sediment was washed by 1 ml sterile saline, and 50ul TB lysate buffer was added for re-suspension. The re-suspended solution was treated by ultrasonic (power 300 W) for 15 min. 2ul of the treated solution was added into a micro reaction tube containing 30ul of amplification detection solution. The micro reaction tube was placed on a dry heat thermostat (60 °C) for 10 min, and then placed at 42 °C for 5 min, with the reaction enzyme being preheated to 42°Cat the same time. Finally, 10ul of enzyme was added to each reaction tube, was quickly mixed and transferred to a real-time fluorescence quantitative PCR instrument. Reaction procedure: The fluorescence channel was set to FAM, and each cycle was at 42 °C for 1 min, with a total of 40 cycles; the fluorescence signal was collected once per minute, and 40 times as a total. Positive result criterion: The abscissa reading (detection time) of the intersection of the sample curve and the threshold line is < 40 min. Negative and positive controls must be done for each test.

### MTB identification

MTB identification was performed if the results of culture and TB-SAT were inconsistent. Take the last third re-suspended solution and perform DNA extraction and identification of MTB complexes.

### Mycobacterial species identification

Mycobacterial species identification was performed for culture-positive but TB-SAT-negative samples according to the mycobacterial species identification test kit instructions. The reverse dot hybridization method was used to identify mycobacteria species. The reference strain of MTB (H37Rv) was obtained from the National Tuberculosis Reference Laboratory of the Chinese Center for Disease Control and Prevention.

### Statistical analysis

SPSS 13.0 analysis software was used for statistical analysis. The sensitivity, specificity, positive predictive value and negative predictive value of the SAT method were compared with the culture method and clinical diagnosis of the patient. The positive rate of MTB in the culture method and SAT test was compared with Chi-square test. *P* < 0.05 was considered statistically significant.

## Results

### Comparison of TB-SAT and culture methods

Four hundred eighty-two extra-pulmonary tuberculosis specimens tested by the TB-SAT and culture methods. The results were displayed in the Table 1. The positive rate of the culture methods was 26.6% (128/482) and the positive rate of TB-SAT was 37.3% (180/482). The positive rate of culture methods and TB-SAT were 29.3% (127/433) and 41.6% (180/433) in the Group A, respectively. The positive rate of the culture methods and TB-SAT were 4.1% (1/49) and 0% (0/49) in the Group B, respectively. The difference in the positive rates of TB-SAT and culture method was statistically significant (χ2 = 106.11, *P* < 0.05). Using culture method as the gold standard the results of sensitivity, specificity, positive predictive value and negative predictive value were displayed in the Table 1. Using clinical diagnosis as the standard, the results of sensitivity, specificity, positive predictive value and negative predictive value were displayed in the Table 2. The difference in the positive rates of TB-SAT and clinical diagnosis was statistically significant (χ2 = 27.67, *P* < 0.05) and the positive rates of culture method and clinical diagnosis was statistically significant (χ2 = 13.06, *P* < 0.05).

**Table 1:** Performances of SAT-TB and culture methods with clinical specimens (*n* = 482)

SAT-TB result	No. of specimens with culture results		Mean %			
	Positive	Negative	Sensitivity	Specificity	PPV	NPV
All samples			83.6%	79.4%	59.4%	93.0%
Positive	107	73				
Negative	21	281				
Group A			84.3%	76.1%	59.4%	92.1%
Positive	107	73				
Negative	20	233				
Group B			0%	100%	100%	98.0%
Positive	0	0				
Negative	1	48

**Table 2:** Performances of SAT-TB and culture methods compared with clinical assessment of patients (*n* = 482)

		No. of cases with clinical diagnosis of TB		Mean %			
Method	Result	Positive	Negative	Sensitivity	Specificity	PPV	NPV
SAT-TB assay				41.6%	100%	100%	16.2%
	Positive	180	0				
	Negative	253	49				
Culture methods				29.3%	98.0%	99.2%	13.6%
	Positive	127	1				
	Negative	306	48				

### MTB identification results

Among the 482 samples, results of 94 culture method were inconsistent with the results of the TB-SAT test. Of 94 samples, 73 samples were positive TB-SAT test results and negative culture results; 21 samples were negative TB-SAT test results and positive culture results. MTB identification was used on all the 94 strains. Of 73 samples the results of MTB identification were 70 positive results and 3 negative results; of the rest 21 samples the results of MTB identification were 3 positive results and 18 negative results. The details of results were displayed in the Table 3.

**Table 3:** Results of MTB identification of samples that TB-SAT results were inconsistent with culture methods (*n* = 94)

Groups	No. of samples	Results		
		Culture methods	TB-SAT	MTB
Group A	3	+	–	+
	17	+	–	–
	70	–	+	+
	3	–	+	–
Group B	1	+	–	–

“+” Presents “Positive”, “-” Presents “Negative”

### Identification of mycobacteria species

Twenty-one strains with negative TB-SAT results and positive culture results were identified. Mycobacterial species identification results showed that 3 cases were *Mycobacteria Intracellular*, 1 case was *Mycobacterium Cheloniae Abscess Subspecies* and 1 case was *Mycobacterium Gordon’s*. The remaining 16 strains were MTB. The details were shown in Table 4.

**Table 4:** Results of mycobacterium species identification (*n* = 21)

Groups	No. of samples	Results			
		Culture methods	TB-SAT	MTB	Species identification
Group A	3	+	–	+	MTB
	13	+	–	–	MTB
	2	+	–	–	**Intracellular** mycobacterium
	1	+	–	–	Gordon’s mycobacterium
	1	+	–	–	Mycobacterium cheloniae abscess subspecies
Group B	1	+	–	–	**Intracellular** mycobacterium

“+” Presents “Positive”, “-” Presents “Negative”

## Discussion

Simultaneous Amplification and Testing (SAT) is a nucleic acid detection technology. The nucleic acid isothermal amplification and real-time fluorescence detection are combined in this technology. The principle is that MTB 16S rRNA was reverse transcribed using Moloney murine leukemia virus (M-MLV) reverse transcriptase in order to generate a 170-bp DNA fragment using a pair of specific primers incorporating the T7 promoter sequence in the sense primer. The DNA was transcribed to RNA by using T7 RNA polymerase to undergo successive cycles of amplification. An internal labeled probe was included and released a fluorescence signal when hybridized with the target RNA. The dynamic measurements of the real-time amplification fluorescence signal were detected using a real-time PCR instrument. This technology has been widely used in the clinical detection of MTB in sputum for the early diagnosis of tuberculosis, but the clinical value for the detection of EPTB is rarely reported.

Culture of M. tuberculosis is the gold standard for diagnosis of active MTB from clinical samples. In this study the Roche method, Bactec MGIT 960 liquid culture method and SAT method were used simultaneously to detect MTB of EPTB specimens. Using the culture methods as the gold standard, the sensitivity and specificity of TB-SAT detection were 83.6 and 79.4%, respectively, and the agreement of the two methods was 80.5%. Most literature reports SAT applied to the detection of MTB sensitivity in sputum in 21.2–75.8% and specificity in 94.73–100% [4–7, 9]. Fan Lin et al. believed that the differences are related to the treatment of sputum specimens, transport and focal pulmonary tuberculosis, not with the method itself. Using clinical diagnosis as the gold standard, the sensitivity and specificity of the TB-SAT detection were 41.6 and 100% in our study, respectively, which was slightly higher than the culture methods whose sensitivity and specificity were 29.3 and 98.0%, respectively. This was mainly due to the positive results of SAT detection could exclude single infection of non-tuberculosis mycobacterium (NTM). However, SAT could not detect mixed infection of MTB and NTM in the same sample. Additionally, the detection target of SAT-TB method is rRNA. RNA products are much more labile outside the reaction tube than DNA amplification products. Base on the higher specificity of SAT test that can generally be completed within 2 h, when both the SAT and sputum smears are positive, it is most likely for the patient to be infected with the MTB. Although, in this study, the negative predictive value of SAT was low, suggesting that clinicians cannot exclude tuberculosis based on the negative SAT test result and still need to use other test results to confirm the diagnosis.

Roche culture method is a traditional and classical laboratory method in the detection of mycobacteria and has long been considered as the gold standard for the diagnosis of tuberculosis, and its test results are usually used as the gold standard for evaluating new MTB detection reagents. Taking into account the mycobacterium vitality and Roche’s nutrient status of roche medium, this study used Roche culture method and Bactec MGIT 960 liquid culture method to detect specimens at the same time in order to improve the sensitivity of the culture method detection results. Bactec MGIT 960 liquid culture method was contrast with SAT test and Roche culture method was used identified the samples with discordant results between the Bactec MGIT 960 liquid culture and SAT-TB methods. The real-time fluorescent PCR method has high sensitivity and specificity for detection of MTB nucleic acid. When the results of SAT detection and culture method are inconsistent, another real-time fluorescent PCR detection reagent is used for evaluation. There was also a large cohort study to further analyze the specificity of the SAT detection method [10]. When the test results are inconsistent, a repeat test of SAT is performed with a detection sensitivity of 75.8%. In this study, 94 specimens with inconsistent TB-SAT and culture methods were tested for MTB identification. The agreement between TB-SAT and real-time fluorescence PCR was 92.6% (87/94). In order to confirm the consistency among the methods, 21 strains of TB-SAT-negative and culture-positive strains were tested for mycobacterial species identification in this study. The results showed that only 5 cases were NTM and the other 16 cases were MTB accounted for 76.2% (16/21), which means that patients with SAT-negative and culture-positive patients should not be considered clinically infected with MTB. Three cases with negative TB-SAT test, positive cultures results and positive MTB assays were analyzed for possible reasons: impurities in the samples affecting TB-SAT amplification reaction, or special components in non-sputum samples (such as pus, tissue, etc.) leads to uneven distribution of target in the sample, or less bacteria in the sample and uneven distribution of template in the lysate, etc. 73 cases with negative culture method and TB-SAT positive samples were identified by PCR. The results showed that 70 cases of TB-SAT and PCR were positive, accounting for 95.9% (70/73). The other 3 cases were culture negative and TB-SAT positive. Samples that were negative by PCR were not confirmed by further tests. This was a deficiency of this study. In addition SAT detection was also an asset in early detection and management of PTB suspects, especially in those patients who are smear negative or sputum scarce and low cost and rapidity [9, 10].

As same as all detection methods, there are some limitations of SAT for detection of EPTB. The first is the pretreatment of EPTB sample that is different from PTB. Some EPTB samples need more time to be processed such as bone, lymph node and so on. The second is RNA degradation. The principle of SAT is that MTB 16S rRNA was reverse transcribed using Moloney murine leukemia virus (M-MLV) reverse transcriptase in order to generate a 170-bp DNA fragment using a pair of specific primers incorporating the T7 promoter sequence in the sense primer. The DNA was transcribed to RNA by using T7 RNA polymerase to undergo successive cycles of amplification. If large RNA degraded quickly, the result may not be right. The Third is the number of live MTB in the EPTB sample. Enough live MTB can produce a large of RNA for detection. The lack of live MTB may affect detection results. In view of the above three limitations EPTB sample should be processed quickly and correctly in order to improve the results of SAT detection.

In summary, SAT has the advantages of stable reaction, high sensitivity and specificity, simple reaction conditions, simple operation, no temperature cycling, high amplification efficiency, and short reaction time; RNA amplification products and templates are both RNA and RNA are easily degraded and laboratory contamination can be greatly reduced. SAT-TB detection is a simple, rapid, sensitive and specific tuberculosis detection method. It can be used clinically to assist in the diagnosis of EPTB and at the same time to some extent increase the positive rate of EPTB patients. SAT-TB is a higher clinical diagnosis value for EPTB in clinical microbiology laboratories.

## Conclusion

SAT test is a simple and rapid test with high specificity for the detection of EPTB. SAT-TB is a higher clinical diagnosis value for EPTB in clinical microbiology laboratories.

## Data Availability

The data set generated and/or analyzed during the current study are not publicly available due to non-disclosure agreements with data providers, but are available from the corresponding author on reasonable request.

## Abbreviations

DNA: Deoxyribonucleic acid
EPTB: Extra-pulmonary tuberculosis
MGIT: Mycobacterium Growth Indicator Tube
M-MLV: Moloney murine leukemia virus
MTB: *Mycobacterium tuberculosis*
NMTB: Non *Mycobacterium tuberculosis*
NTM: Non-tuberculosis mycobacterium
PCR: Polymerase chain reaction
RNA: Ribonucleic acid
rRNA: ribosomal ribonucleic acid
SAT: Simultaneous amplification and testing
SAT-TB: Simultaneous amplification and testing tuberculosis
TB: Tuberculosis
TB-SAT: Tuberculosis simultaneous amplification and testing
WHO: World Health Organization
